# Trajectories of Health Changes in Older Adults with Chronic Hepatitis B Infection

**DOI:** 10.1093/geroni/igaa057.3331

**Published:** 2020-12-16

**Authors:** I-Chien Wu, Chang-Hua Chen, Yu-Wen Yang, Yen-Tze Liu, Yen-Feng Chiu, Chih-Cheng Hsu, Shu-Chun Chuang, Chao A Hsiung

**Affiliations:** 1 National Health Research Institutes, Taiwan, Zhunan, Miaoli County, Taiwan (Republic of China); 2 Changhua Christian Hospital, Changhua City, Taiwan, Changhua, Taiwan (Republic of China)

## Abstract

Despite the increasing burden of chronic hepatitis B (CHB) in aging populations, little is known about the course of health-related quality of life (HRQoL) changes during older adulthood in CHB patients. We aimed to assess individual-level longitudinal HRQoL changes in older patients with CHB and to examine their correlates. A 5-year prospective cohort study was conducted in 503 hepatitis B surface antigen-positive community-dwelling adults aged 55 years or older. Participants underwent comprehensive assessments at baseline and serial measurement of HRQoL using the short-form (12) health survey version 2. Of these participants, 82.7% remained in good physical health throughout the study period, whereas 9.1% had declining physical health and 8.2% were in poor physical health. We likewise identified three trajectories of mental health changes (“good mental health” [86.9%], “declining mental health” [6.8%], and “poor mental health” [6.4%]). Three baseline characteristics were independently associated with a lower likelihood of remaining physically or mentally healthy during older adulthood: sarcopenic obesity (odds ratio [OR] with 95% confidence interval [95% CI] of 7.5[2.8-20.5] for poor physical health, 3.1 [1.1-8.4] for declining physical health, 4.3 [1.4-13.0] for poor mental health), higher number of metabolic abnormalities (OR [95% CI] of 3.6 [1.6-8.0] for poor physical health) and depressed mood (OR [95% CI] of 21.7 [5.8-81.0] for poor physical health, 5.3 [1.4-19.9] for declining physical health, 83.1 [19.7-350.2] for poor mental health, 13.6 [2.9-64.8] for declining mental health). In conclusion, we demonstrated the heterogeneity and nonlinearity of HRQoL changes and their associations with variations in specific extrahepatic organs/systems.

